# Identification of CD8+ T cell host factors involved in HIV control

**DOI:** 10.1186/1742-4690-9-S2-O44

**Published:** 2012-09-13

**Authors:** G Gaiha, K McKim, M Woods, M Lichterfeld, A Brass, B Wallker

**Affiliations:** 1 Ragon Institute of MGH, MIT and Harvard/Massachusetts General Hospital, Boston, MA, USA

## Background

The goal of the is project was to further define our understanding of the critical antiviral effector molecules and cell signaling pathways involved in CD8 T cell mediated control of HIV infection.

## Methods

To accomplish this, we performed whole genome transcriptional profiling on sorted epitope-specific CD8 T cells, in the presence and absence of cognate peptide, from a total of 8 B*2705 elite controllers and progressors. Epitope-specific CTLs were isolated using HLA class I tetramers and used to prepare mRNA for evaluation by gene expression profiles using whole genome Illumina bead arrays. Bulk CD8 T cells were used as an internal control for each patient.

## Results

Statistically significant genes enriched by our analysis include those involved in cellular cytotoxicity, cytokine signaling, T-cell receptor signaling, cell-cell adhesion molecules, and T cell homeostasis. Further analysis of the gene set identified 137 unique genes that were both induced by peptide stimulation in epitope-specific CD8 T cells, and significantly different between peptide-stimulated controller and progressor CD8 T cells. Further refinement of this list of 137 genes using a protein-protein network analysis revealed that 13 of these genes are statistically significant in driving the network connectivity. We have now focused our efforts on one of these genes, Caspase 8.

## Conclusion

This study defines a novel set of 137 genes in epitope-specific CD8 T cells that are upregulated upon peptide stimulation and different between patients that control and have progressive infection. Further refinement of this list defines 13 unique candidates that drive network connectivity, of which one was Caspase 8. Our initial studies also reveal that Caspase 8 mRNA expression, which has a known role in T cell homeostasis, is upregulated in CD8 T cells in controllers, suggesting its potential role as a novel T cell correlate of control.

